# P-2034. Inappropriate Outpatient Antibiotic Prescribing for COVID-19 in New York City, 2020—2022

**DOI:** 10.1093/ofid/ofae631.2190

**Published:** 2025-01-29

**Authors:** Mary K Foote, Nisha Kochar, Nang Thu Thu Kyaw

**Affiliations:** New York City Department of Health and Mental Hygiene, Brooklyn, New York; New York City Department of Health and Mental Hygiene, Brooklyn, New York; New York City Department of Health and Mental Hygiene/Centers for Disease Control and Prevention, Long Island City, NY, New York

## Abstract

**Background:**

Antibiotic misuse is a key driver of antimicrobial resistance. Early in the COVID-19 pandemic, several unproven therapies were used, including antibiotics. In November 2020 and December 2021, monoclonal antibodies and oral antivirals were authorized for the outpatient treatment for COVID-19 respectively, which may have influenced inappropriate antibiotic prescribing (IAP). Here we describe prescribing trends and proportion of inappropriate antibiotic prescriptions for COVID-19 by patient and provider characteristics during 2020—2022 in New York City (NYC).

**Figure:**
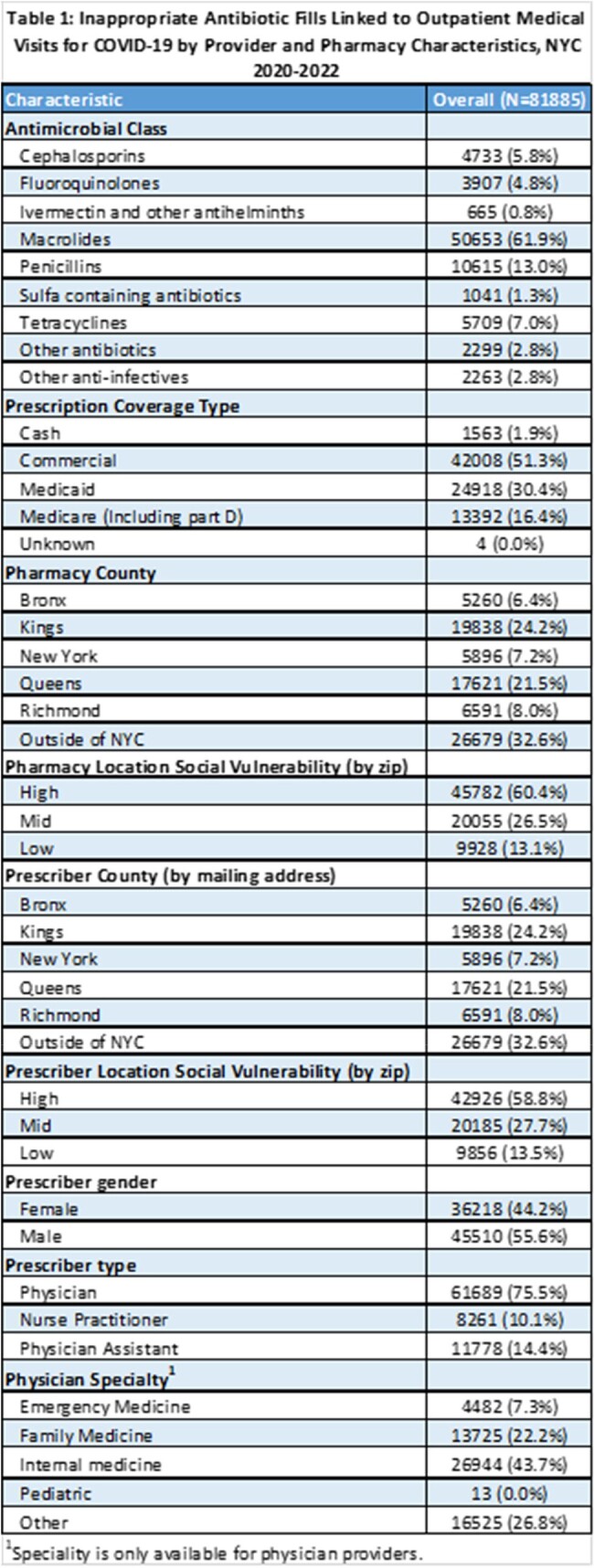

**Methods:**

We performed a retrospective descriptive analysis of medical visits for COVID-19 during 2020—2022 identified by ICD-10 codes and associated antibiotic prescription fills using IQVIA medical data, which captures office-based physician and specialists visit claims, and prescription data, which captures 94% of retail pharmacy prescription claims. Medical visits with a concomitant diagnosis for conditions which may or do require antibiotics were excluded. Inappropriate antibiotic prescription (IAP) was defined as an antibiotic prescription claim linked to a COVID-19 medical visit within ≤7 days.

**Figure:**
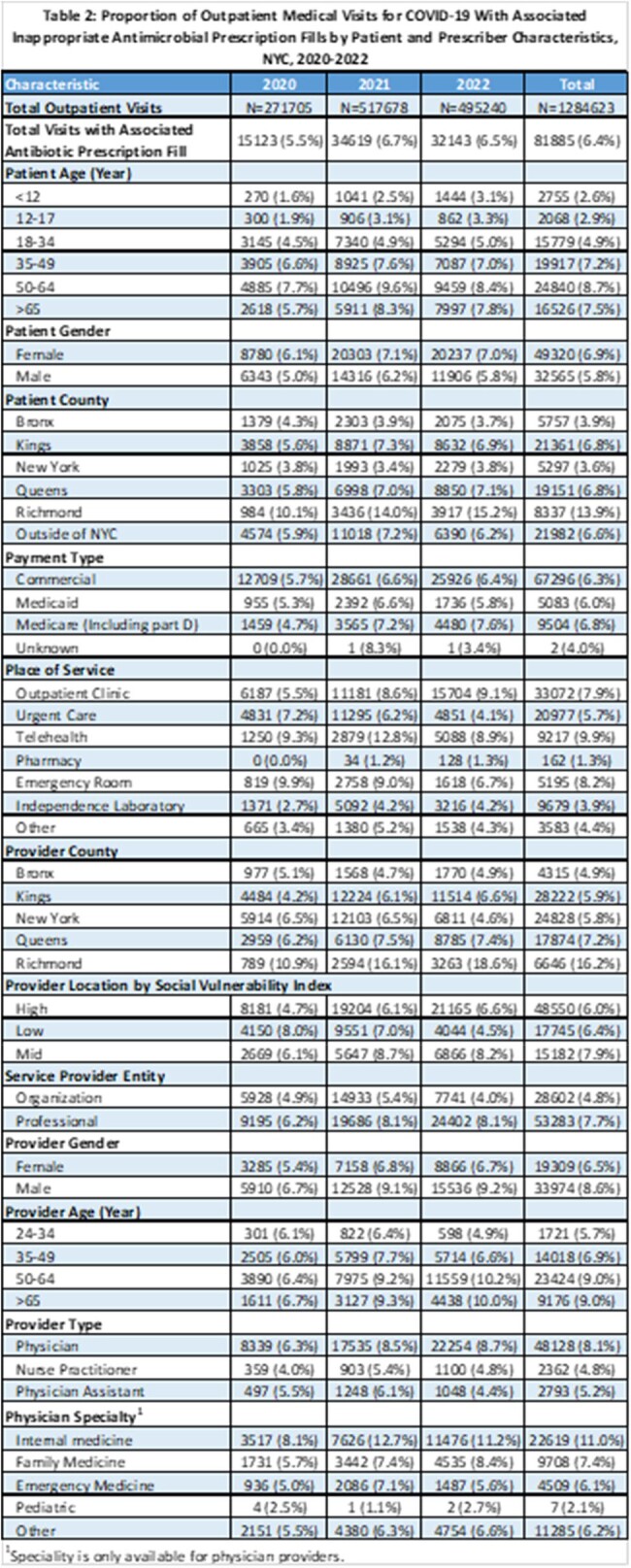

**Results:**

Of 1,284,623 medical visits for COVID-19, 81,885 (6.4%) resulted in an antibiotic prescription fill, a majority were macrolides (62%). Most patients were seen in a clinic (32.4%) or urgent care (28.7%) and by a physician (85.2%), most frequently Internal Medicine (34.7%). Rates of IAPs were 5.5% in 2020, 6.7% in 2021 and 6.5% in 2022. Patients aged 50-64 years (8.7%), who were female (6.9%) or had a telehealth encounter (9.9%) received more IAPs than other groups. Antibiotics were more likely to be inappropriately prescribed by providers who were male (8.6%), aged ≥50 years (7.6%) or internal medicine physicians (9.0%).

**Conclusion:**

Availability of COVID-19 treatments did not appear to decrease antibiotic prescribing. IAP was lower overall compared to national estimates but varied significantly by patient and provider characteristics. Limitations include lack of data on unfilled prescriptions, race, ethnicity, disease severity or underlying conditions. These findings can help target interventions to improve quality of treatment for COVID-19.

**Disclosures:**

All Authors: No reported disclosures

